# Supplementary material for: Quality of Life Identifies High-Risk Groups in Advanced Rectal Cancer Patients
Source: Healthcare (Basel). 2025 Jul 23;13(15):1782. doi: 10.3390/healthcare13151782 (PMC12345726; doi:10.3390/healthcare13151782)
Supplement: Supplementary file 1 [file healthcare-13-01782-s001.zip › Suppl. table S1_p-values.pdf]

	Day 1	Week 2	Week 5	Week 10	Year 1	Year 2	Year 3	Year 4	Year 5
**EORTC QLQ-30**									
Functional scales:									
Physical functioning	<0,001	<0,001	0,075	<0,001	<0,001	<0,001	<0,001	<0,001	<0,001
Role functioning	<0,001	0,085	0,458	0,003	<0,001	<0,001	<0,001	0,003	<0,001
Emotional functioning	0,423	0,066	0,133	0,045	<0,001	0,002	<0,001	0,007	0,002
Cognitive functioning	0,036	0,113	0,485	0,234	0,006	0,022	0,041	0,005	0,003
Social functioning	0,365	0,031	0,383	0,156	<0,001	0,002	0,006	0,001	<0,001
Global QoL/GHS	0,011	0,012	0,130	0,001	<0,001	<0,001	<0,001	<0,001	<0,001
Symptom scales:									
Fatigue	<0,001	<0,001	0,320	0,014	<0,001	0,002	<0,001	<0,001	0,002
Nausea and vomiting	0,560	0,039	0,120	0,098	0,021	<0,001	<0,001	0,008	0,152
Pain	<0,001	0,019	0,041	0,703	0,037	0,028	0,011	<0,001	0,412
Single items:									
Dyspnoea	0,002	0,004	0,293	0,010	<0,001	<0,001	<0,001	0,004	<0,001
Sleep disturbance	0,745	0,012	0,420	0,240	0,017	0,104	<0,001	0,014	0,017
Appetite loss	0,010	0,002	0,565	0,120	0,003	0,013	<0,001	0,009	<0,001
Constipation	0,297	0,238	0,228	0,021	0,032	0,793	0,127	0,379	0,821
Diarrhoea	0,319	0,305	0,834	0,023	0,518	0,243	0,258	0,608	0,149
Financial difficulties	0,213	0,861	0,081	0,156	0,530	0,569	0,267	0,768	0,820
**EORTC QLQ-CR38**									
***Functional scales*:**									
Body image	0,173	0,161	0,130	0,261	0,008	0,800	0,290	0,011	0,055
Future perspective	0,469	0,335	0,276	0,260	0,016	0,250	0,006	0,012	0,144
Sexual functioning	<0,001	<0,001	0,034	<0,001	<0,001	0,014	0,013	0,003	0,514
Sexual enjoyment	<0,001	0,041	0,703	<0,001	0,838	0,183	0,924	0,314	0,767
Symptom scales:									
Micturition problems	0,412	0,030	0,118	0,201	<0,001	0,002	0,001	0,011	0,098
Gastrointestinal tract symptoms	0,298	0,083	0,008	0,202	0,086	0,256	0,076	0,252	0,303
Chemotherapy side-effects	0,002	0,007	0,077	0,023	<0,001	<0,001	<0,001	0,070	0,009
Problems with defacation	0,044	0,006	0,243	0,028	0,090	0,257	0,025	0,009	0,607
Stoma-related problems	0,101	0,059	0,005	0,006	0,243	0,156	<0,001	0,114	0,348
Male sexual problems	0,036	0,014	0,034	0,007	0,294	0,092	0,396	0,217	0,495
Female sexual problems	0,640	0,437	0,317	0,051	0,505	0,617	0,739		
Weight loss	0,006	0,023	0,619	0,098	0,039	0,004	0,003	0,018	0,266
