# Supplementary material for: Quality of Life Identifies High-Risk Groups in Advanced Rectal Cancer Patients
Source: Healthcare (Basel). 2025 Jul 23;13(15):1782. doi: 10.3390/healthcare13151782 (PMC12345726; doi:10.3390/healthcare13151782)
Supplement: Supplementary file 1 [file healthcare-13-01782-s001.zip › Suppl. Table S2_Multivariate analysis of Role functioning, Dyspnoea, Chemotherapy side effects.pdf]

Suppl. table S2. Univariate and multivariate analysis of overall survival (Cox proportional hazard's model) comparing patients with at least two better scores vs. at least two poor scores

Variable	HR	95% C.I.	p	HR	95% C.I.	p
**Role functioning** (poor [n = 119] v. better [n = 239])	0.663	0.449 – 0.979	0.039	0.641	0.423 – 0.970	0.036
Age, years (under 64 years) [n = 283] v. over 64 years [n = 268])	1.864	1.398 – 2.484	<0.001	1.995	1.322 – 3.012	0.001
T category (T1/T2/3 [n = 410] v. T4 [n = 141])	1.489	1.097 – 2.020	0.011	1.283	0.818 – 2.013	0.278
N category (N0 [n = 164] v. N+ [n = 380])	0.941	0.692 – 1.279	0.696	---	---	---
M category (M0 [n = 412] v. M+ [n = 86])	2.429	1.751 – 3.368	<0.001	2.579	1.642 – 4.051	<0.001
gender (male [n = 389] v. female [n = 162])	1.017	0.745 – 1.388	0.916	---	---	---

Variable	HR	95% C.I.	p	HR	95% C.I.	p
**Side effects of chemotherapy** (less [n = 126] v. more [n = 192])	0.567	0.387 – 0.833	0.004	0.626	0.413 – 0.949	0.027
Age, years (under 64 years) [n = 283] v. over 64 years [n = 268])	1.864	1.398 – 2.484	<0.001	2.471	1.624 – 3.759	<0.001
T category (T1/T2/3 [n = 410] v. T4 [n = 141])	1.489	1.097 – 2.020	0.011	1.204	0.765 – 1.895	0.422
N category (N0 [n = 164] v. N+ [n = 380])	0.941	0.692 – 1.279	0.696	---	---	---
M category (M0 [n = 412] v. M+ [n = 86])	2.429	1.751 – 3.368	<0.001	2.741	1.742 – 4.312	<0.001
gender (male [n = 389] v. female [n = 162])	1.017	0.745 – 1.388	0.916	---	---	---

Variable	HR	95% C.I.	p	HR	95% C.I.	p
**Dyspnoea** (poor [n = 52] v. better [n = 309])	0.448	0.295 – 0.679	<0.001	0.444	0.286 – 0.692	<0.001
Age, years (under 64 years) [n = 283] v. over 64 years [n = 268])	1.864	1.398 – 2.484	<0.001	2.456	1.664 – 3.624	<0.001
T category (T1/T2/3 [n = 410] v. T4 [n = 141])	1.489	1.097 – 2.020	0.011	1.539	1.028 – 2.303	0.036
N category (N0 [n = 164] v. N+ [n = 380])	0.941	0.692 – 1.279	0.696	---	---	---
M category (M0 [n = 412] v. M+ [n = 86])	2.429	1.751 – 3.368	<0.001	3.141	2.025 – 4.873	<0.001
gender (male [n = 389] v. female [n = 162])	1.017	0.745 – 1.388	0.916	---	---	---
