# Supplementary figures and images for: Quality of Life Identifies High-Risk Groups in Advanced Rectal Cancer Patients
Source: Healthcare (Basel). 2025 Jul 23;13(15):1782. doi: 10.3390/healthcare13151782 (PMC12345726; doi:10.3390/healthcare13151782)

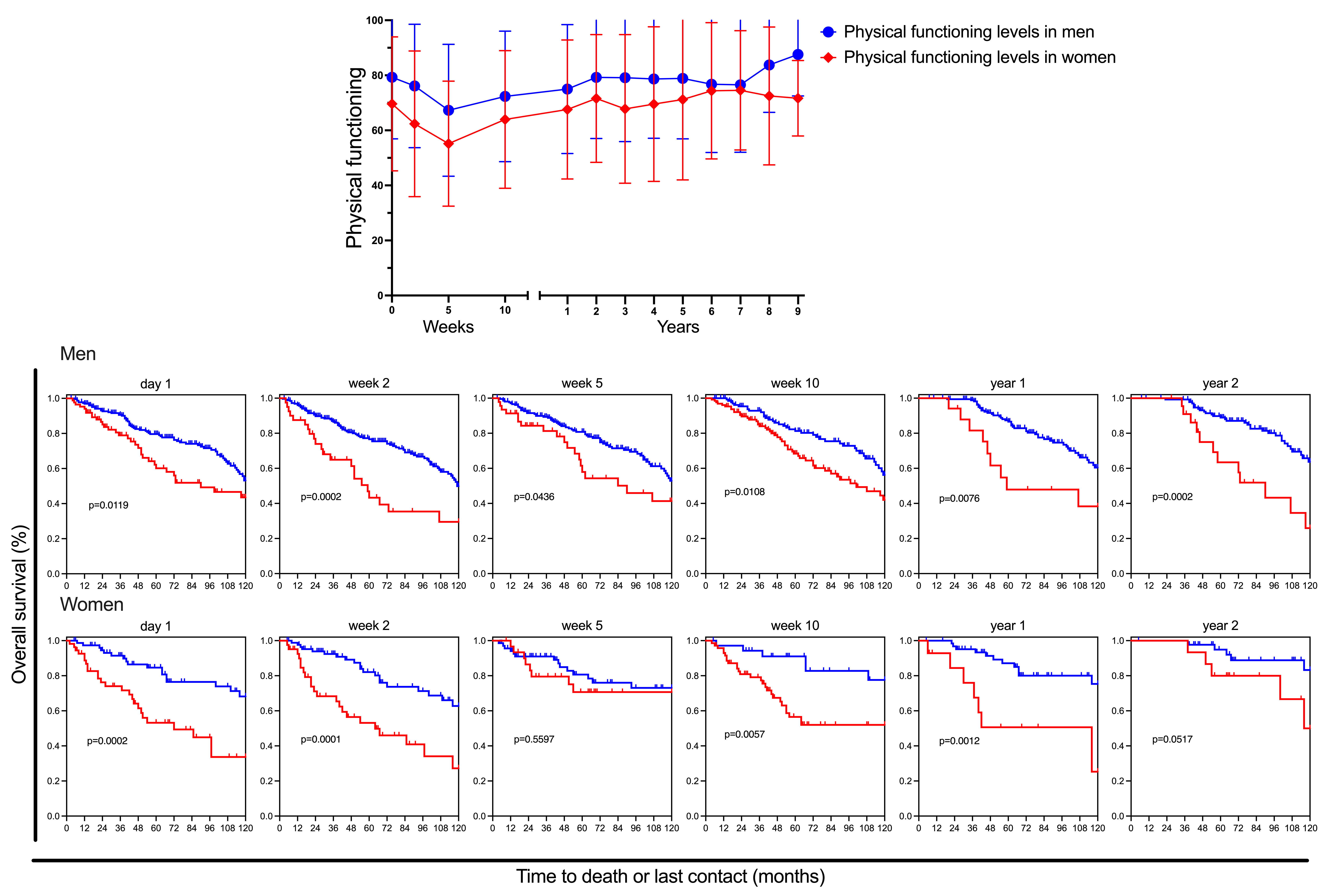

Supplement: Supplementary file 1 [file healthcare-13-01782-s001.zip › Suppl. fig. S2_ sex difference physical functioning.png]

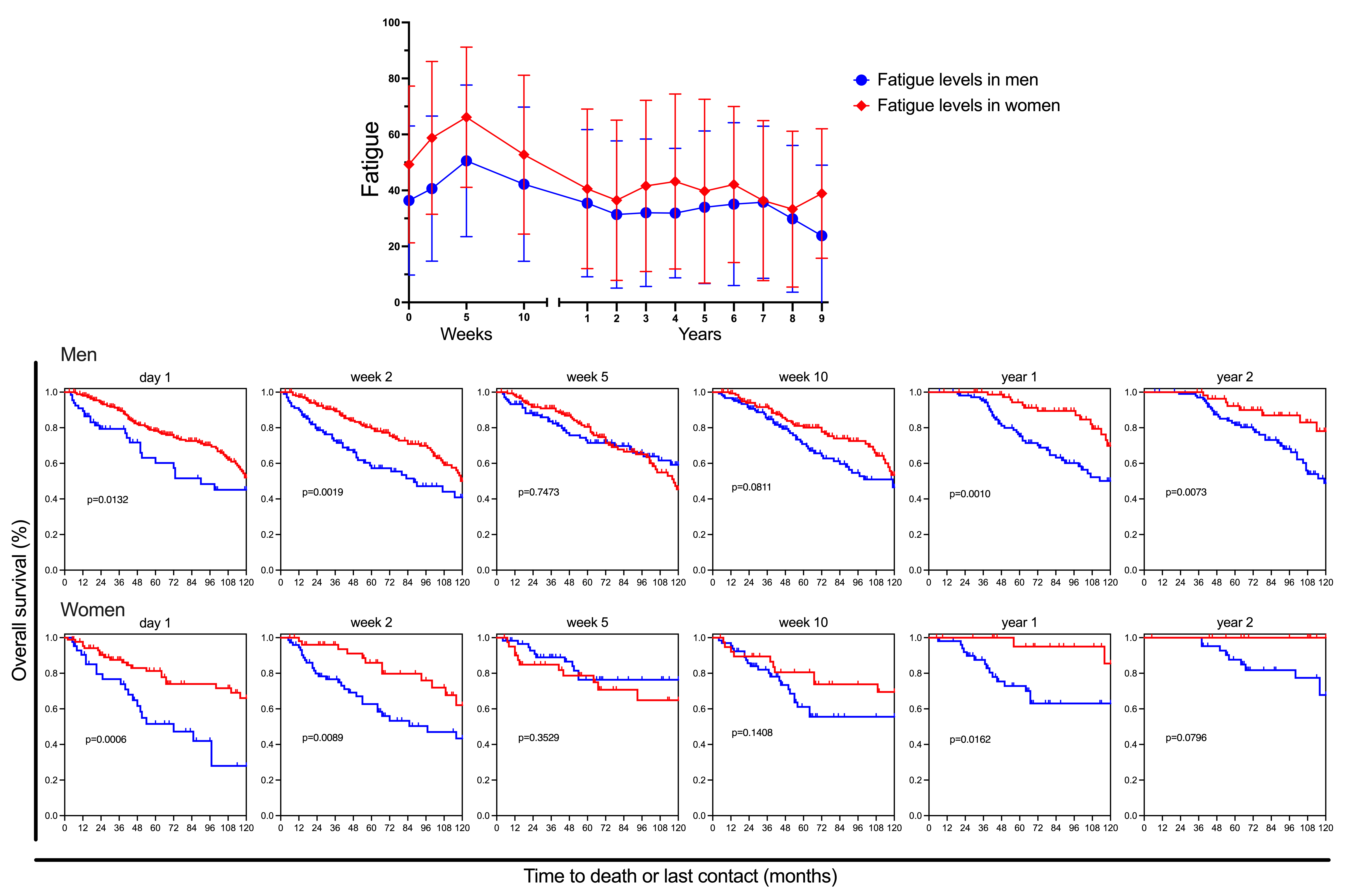

Supplement: Supplementary file 1 [file healthcare-13-01782-s001.zip › Suppl. fig. S3_ sex difference fatigue.png]

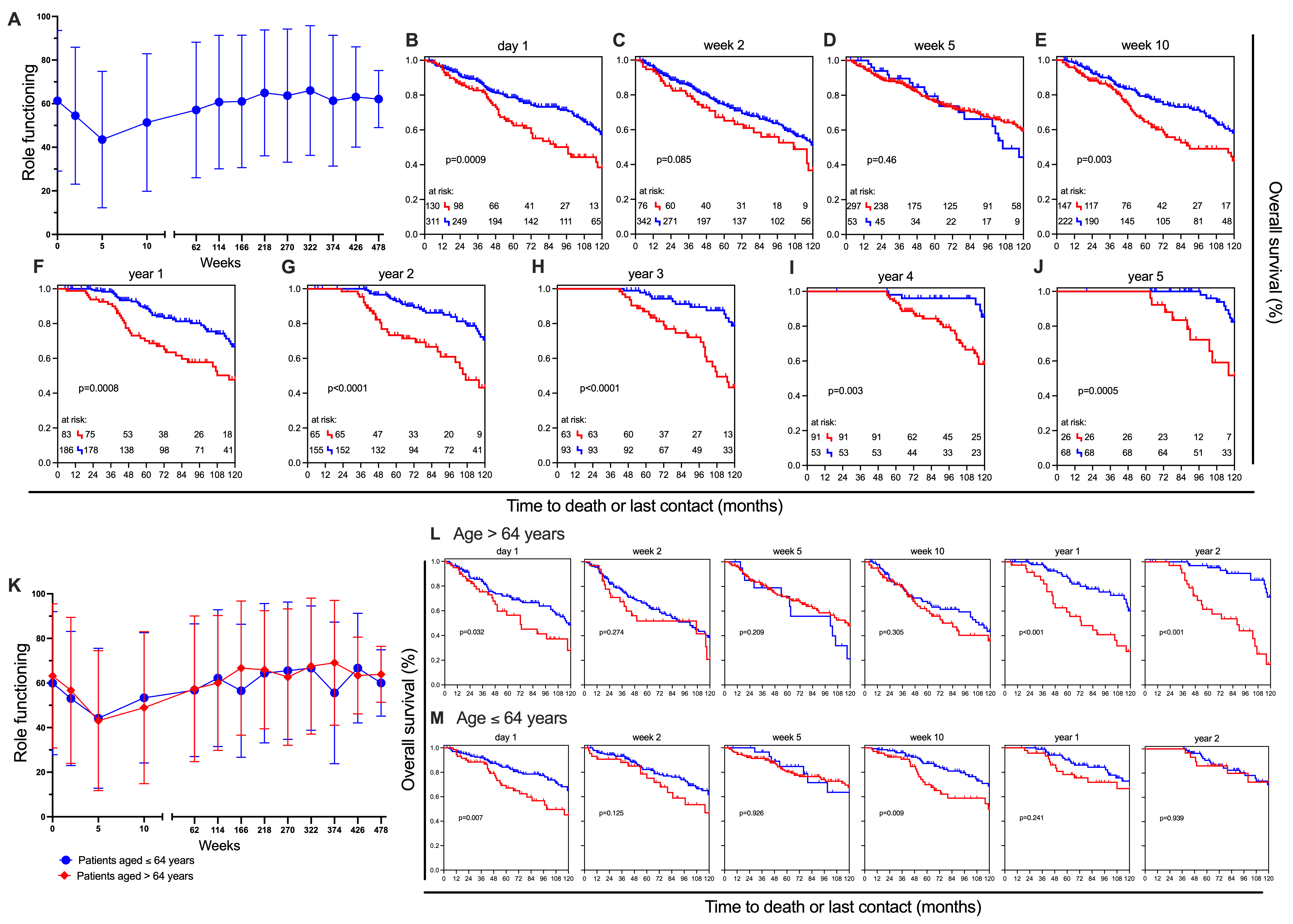

Supplement: Supplementary file 1 [file healthcare-13-01782-s001.zip › Suppl. fig. S4_Role Functioining.png]

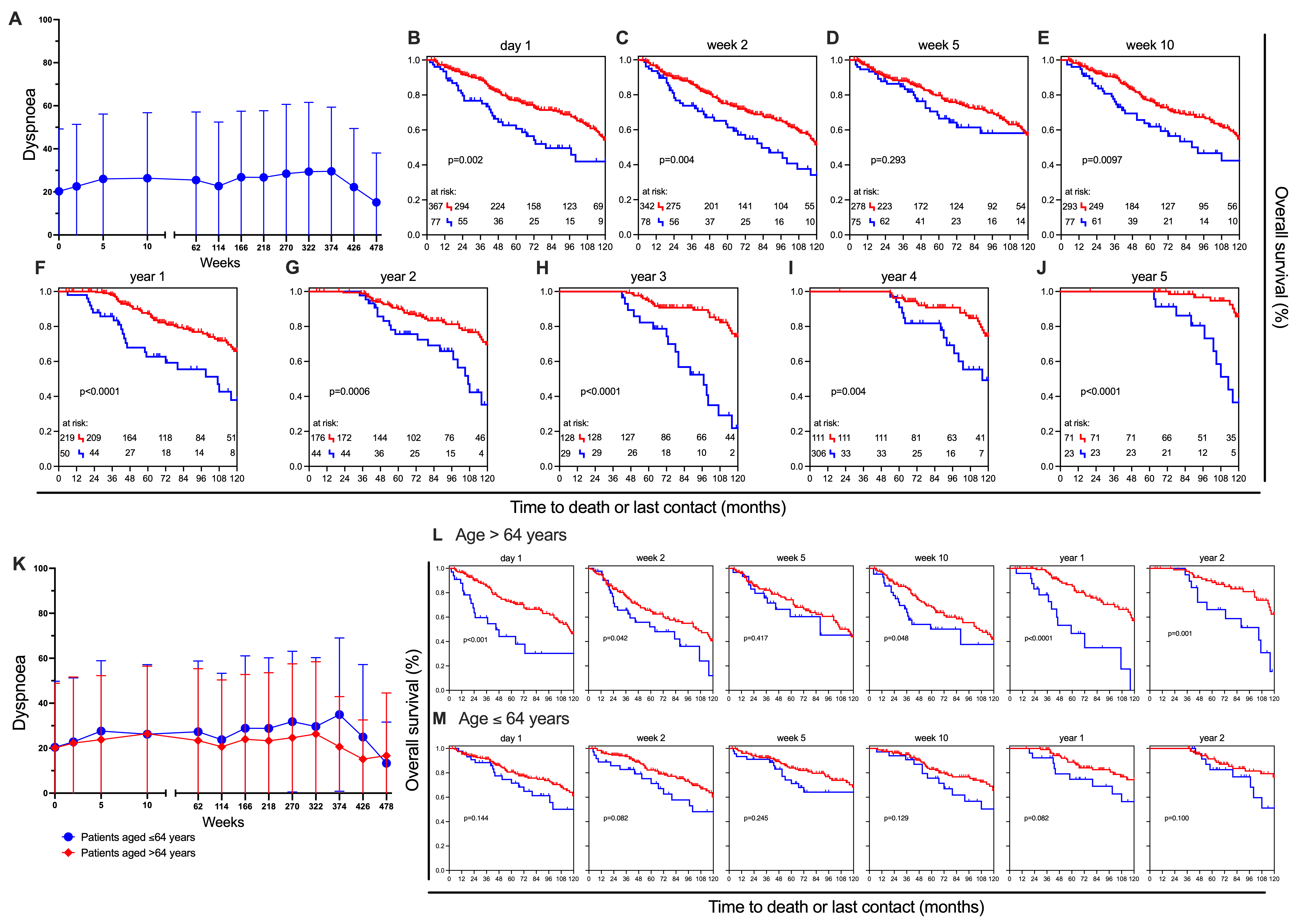

Supplement: Supplementary file 1 [file healthcare-13-01782-s001.zip › Suppl. fig. S5_Dyspnoea.png]

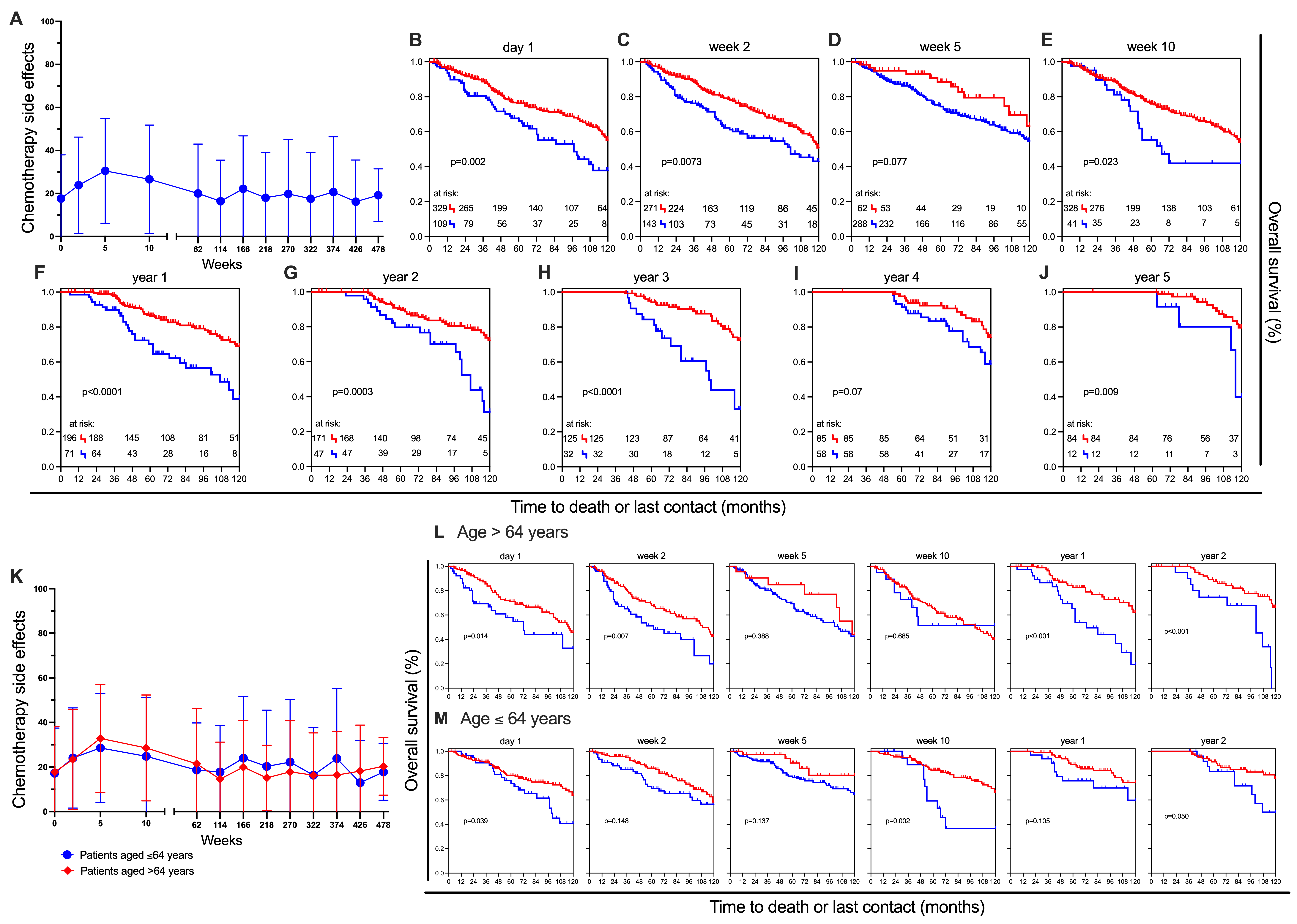

Supplement: Supplementary file 1 [file healthcare-13-01782-s001.zip › Suppl. fig. S6_Chemotherapy side effects .png]
